# Genetic characterization and distribution of the virus in chicken embryo tissues infected with Newcastle disease virus isolated from commercial and native chickens in Indonesia

**DOI:** 10.14202/vetworld.2022.1467-1480

**Published:** 2022-06-14

**Authors:** Liza Angeliya, Yuli Purwandari Kristianingrum, Widya Asmara, Michael Haryadi Wibowo

**Affiliations:** 1 Veterinary Science Doctoral Study Program, Faculty of Veterinary Medicine, Universitas Gadjah Mada, Yogyakarta, 55281, Indonesia; 2 Disease Investigation Center Lampung, Jalan Untung Suropati Bandar Lampung, Lampung, 35142, Indonesia; 3 Department of Pathology, Faculty of Veterinary Medicine, Universitas Gadjah Mada, Yogyakarta, 55281, Indonesia; 4 Department of Microbiology, Faculty of Veterinary Medicine, Universitas Gadjah Mada, Yogyakarta, 55281, Indonesia

**Keywords:** fusion, gene characterization, hemagglutinin-neuraminidase, Newcastle disease, pathogenicity, viral distribution

## Abstract

**Background and Aim:**

Newcastle disease (ND) is a viral infectious disease that affects commercial and native chickens, resulting in economic losses to the poultry industry. This study aimed to examine the viral strains circulating in commercial and native chickens by genetic characterization and observe the distribution of Newcastle disease virus (NDV) in chicken embryonic tissue.

**Materials and Methods:**

ND was detected using a quantitative reverse transcription-polymerase chain reaction. Genetic characterization of the fusion (F) and hemagglutinin-neuraminidase (HN) genes from the eight NDVs was performed using specific primers. The sequence was compared with that of other NDVs from GenBank and analyzed using the MEGA-X software. The distribution of NDV in chicken embryos was analyzed based on lesions and the immunopositivity in immunohistochemistry staining.

**Results:**

Based on F gene characterization, velogenic NDV strains circulating in commercial and native chickens that showed varying clinical symptoms belonged to genotype VII.2. Lentogenic strains found in chickens without clinical symptoms were grouped into genotype II (unvaccinated native chickens) and genotype I (vaccinated commercial chickens). Amino acid variations in the HN gene, namely, the neutralization epitope and antigenic sites at positions 263 and 494, respectively, occurred in lentogenic strains. The NDV reaches the digestive and respiratory organs, but in lentogenic NDV does not cause significant damage, and hence embryo death does not occur.

**Conclusion:**

This study showed that velogenic and lentogenic NDV strains circulated in both commercial and native chickens with varying genotypes. The virus was distributed in almost all organs, especially digestive and respiratory. Organ damage in lentogenic infection is not as severe as in velogenic NDV. Further research is needed to observe the distribution of NDV with varying pathogenicity in chickens.

## Introduction

Newcastle disease (ND) is a contagious disease in commercial and native chickens. ND is caused by the Avian paramyxovirus type I (APMV-I) of the *Paramyxoviridae* family. Newcastle disease virus (NDV) is a synonym for APMV-1. This virus has an envelope and a negatively polarized, unsegmented single-stranded Ribonucleic acid (RNA). Viral replication occurs in the cytoplasm. NDV has various genotypes and is classified in to two classes, I and II [1]. Class I NDV isolates are classified into a single genotype containing three sub-genotypes. NDV Class II has been grouped into at least 21 genotypes with many sub-genotypes based on the coding sequence analysis of the complete fusion gene [2]. The pathogenicity of NDV can be analyzed biologically using mean death time (MDT), intracerebral pathogenicity index (ICPI), and intravenous pathogenicity index (IVPI). Molecular pathogenicity can be determined by characterizing the fusion (F) gene at the cleavage site [3, 4]. Velogenic strain NDVs have MDT values <60 h, mesogenic strains 60–90 h, and lentogenic strains >90 h in embryonic chicken eggs. The ICPI value of velogenic strain NDVs was 1.5–2 in 1-day-old chickens. Mesogenic strains showed an ICPI of 1.0–1.5, and lentogenic strains had an average ICPI value close to 0.0 (0.0–0.5) [4]. The IVPI values of velogenic NDVs were in the range of 2.0–3.0, while those of mesogenic and lentogenic strains were 0.0–0.5 and 0.0, respectively [3, 5]. The pathotype and genotype of NDV can be determined by analyzing the F gene sequences encoding the F protein [6]. The F protein has an intersection between F1 and F2, which is called the cleavage site. The amino acid sequence at the cleavage site is a marker for determining the pathogenicity of NDV. Pathogenic NDVs have sequences 112R/KRQR/K-R116 with F (phenylalanine) at position 117, while less pathogenic viruses have sequences 112G/EK/RQG/E-R116 with L (leucine) at position 117 [4]. Interactions between HN and F proteins play a role in infection and virulence [7]. The HN protein, together with F, supports the fusion between the infected cells and surrounding cells. HN also has six glycosylation sites that play important roles in protein folding, stability, and maturation of antigenicity and are determinants of virulence classification [8, 9]. There are many important implications for HN proteins, including glycosylation position, receptor binding, antigenic sites, c-terminal elongation, and neutralization epitopes [10–12].

ND has been widely reported in various hosts with varying genotypes in Indonesia and other countries [13]. The circulating NDV in commercial [14] and native chickens [15], which have been reported to be an infectious velogenic strain, is classified as genotype VII. The lentogenic strain NDV, classified as genotype II, has been reported in native chickens in Bogor, West Java [16], and East Java [17]. This strain has also been found in swan goose [18]. Cases of ND are still reported in both commercial and native chickens in Indonesia, which cause economic losses to the poultry industry. This research is an update of the molecular characterization of F and HN genes that was carried out in Indonesia on NDV isolates originating from commercial chicken farms and native chickens collected in 2019–2020. Moreover, our result showed that NDV genotype II was isolated from un-vaccinated native chicken and genotype I was isolated from broiler breeder that had never been reported in Indonesia. The data of embryonic lesions with IHC staining that comparing velogenic and lentogenic NDV isolates have never been conducted in Indonesia.

This study aimed to examine the viral strains circulating in commercial chicken farms and backyards by characterization of the F and HN genes and to observe the distribution of the virus in chicken embryo tissue that has been infected with the NDV strain circulating in Indonesia.

## Material and Methods

### Ethical approval

This study was approved by the Ethics Commission of the Faculty of Veterinary Medicine, Gadjah Mada University (UGM), Yogyakarta (EC. No:00014/EC-FKH/Int./2021).

### Study period and location

This research was conducted from March to August 2021 at the Microbiology Laboratory of the Faculty of Veterinary Medicine, UGM, Yogyakarta, and the Laboratory of Biotechnology, Virology, and Pathology at the Disease Investigation Center (DIC), Lampung, Indonesia.

### Samples

The number of samples used in this study was eight. Six samples were obtained from pool organs (lungs and spleen) of chickens, that indicated NDV infection. Meanwhile, two samples were prepared from cloacal swabs of chickens, which did not show any symptoms. Chickens showing clinical symptoms included three laying hens with decreased egg production in 30-week-old chickens, a mortality rate of up to 3% in pullets and neurological symptoms in day-old chickens, respectively. Two native chickens and one broiler showed greenish diarrhea, respiratory problems, and died. Meanwhile, one native chicken and one broiler were asymptomatic. Six samples were collected from the working area of DIC Lampung and the other two were collected from the Faculty of Veterinary Medicine UGM.

### Accession numbers

All nucleotide sequences analyzed in this study are available in GenBank. The F and HN gene sequences (n = 16) of the eight NDV (8 F genes and 8 HN genes) obtained in this study were submitted to GenBank and are available under the accession numbers recorded in Table-1.

**Table 1:** History, symptoms, results of qRT-PCR, virus isolation, cleavage site, and accession numbers of the samples in this study.

Sample name	Host Strain	Symptom	Location	Year	qRT-PCR ND (ct)	qRT-PCR AI (ct)	HA titer	MDT (h)	Cleavage site	Accession No. F gene	Accession No. HN gene
Broiler_ISW19	Broiler	Diarrhea and death	Gunung Kidul	2019	24.03	Undet	2^3^	<60	RRQKRF	MZ488460	MZ488469
Broiler_A032009017	Broiler	No symptom	Pesawaran	2020	30.09	Undet	-	>120	GKQGRL	MZ488461	MZ488470
Chicken_P032001007	Native	Diarrhea, death	Bandar Lampung	2020	18.09	Undet	2^7^	<60	RRQKRF	MZ488462	MZ488471
Chicken_BRS20	Native	No symptom	Lampung Tengah	2020	29.24	Undet	2^1^	>120	GRQGRL	MZ488463	MZ488472
Chicken_A031909005	Native	Respiratory symptom, greenish diarrhea, death	Muara Enim	2019	19.77	Undet	2^6^	<60	RRQKRF	MZ488464	MZ488473
Layer_P032006078	Layer	3% of deaths per day	Lampung Selatan	2020	23.96	Undet	2^3^	<60	RRQKRF	MZ488465	MZ488474
Layer_P032006203	Layer	Decreased egg production	Lampung selatan	2020	24.14	Undet	2^3^	<60	RRRKRF	MZ488466	MZ488475
Layer_DOCHTN20	Layer	Nervous symptom	Yogyakarta	2020	32.52	Undet	-	<60	RRQKRF	MZ488467	MZ488476

MDT = Mean death time, qRT-PCR = Quantitative reverse transcription-polymerase chain reaction, Ct = Cycle threshold

### NDV diagnosis using quantitative reverse transcription-polymerase chain reaction (qRT-PCR)

Viral RNA was extracted from eight samples using the Pure Link® Viral RNA/DNA Mini Kit (Invitrogen, Thermo Fisher Scientific, USA) according to the manufacturer’s instructions. ND was diagnosed via qRT-PCR of NDV and avian influenza virus (AIV) matrix genes to determine whether the cause of the disease in chickens was NDV and not AIV as the differential diagnosis. The reagent mix for amplification consisted of the AgPath-ID™ One-Step reverse transcriptase-polymerase chain reaction (RT-PCR) Reagents (Applied Biosystems, Thermo Fisher Scientific, USA), a specific primer set (forward, reverse, and probe primers), and nuclease-free water (NFW) and was added to the extracted viral RNA. The reaction mix was performed according to the manufacturer’s instructions. Primers used for qRT-PCR were one set of primer-probe for M gene of ND (Australian Animal Health Laboratory, Geelong) and one set of primer-probe for M of gene AIV (BBVet Wates). All primers used are listed in Table-2. The solution was amplified using an ABI 7500 Real-Time PCR Thermo Cycler machine, with the following settings: RT (45°C for 10 min) for 1 cycle; pre-denaturation (95°C for 10 min) for 1 cycle; denaturation (95°C for 15 s), and annealing (60°C for 45 s). The results appeared on the monitor screen in the form of curves and cycle threshold (Ct) values. A Ct value <40 was considered a positive result, a Ct value of 40–45 was indeterminate, and a Ct value >45 was negative.

**Table 2:** Primer set for qRT-PCR (AI and ND: M gene) and RT-PCR (ND: F and HN gene).

Primer	Forward	Reverse	Probe
ND_Matrix	5’- ACAACCAAGTGAGGTGAGTACTTG-3’	5’- GACTCCCTTTCTCTGATTGTCCAT-3’	5’FAM-CGTTTCCAGTCGTTGGATTTAC-TAMRA 3’
AI_Matrix	5’ AGATGAGYCTTCTAACCGAGGTCG 3’	5’- TGCAAANACATCYTCAAGTCTCTG-3’	5’FAM-TCAGGCCCCCTCAAAGCCGA-TAMRA 3’
ND_F_Reg1	5’- AGAGTGTGGATCCCAACCAG -3’	5’- GTGGATACAGACCCTTGAATCTTG-3’	-
ND_F_Reg2	5’- GCAGGGATTGTAGTAACAGGAGAT-3’	5’- CCAAGAGTTGAGTCTGTGAGTCAT-3’	-
ND_F_Reg3	5’- ACTACAGTGTTCGGGCCACA-3’	5’- AGCCTCAGAGTTATCCCGTCTAAT-3’	-
ND_F_Reg4	5’- GTTTGAGCGGCAACACATC-3’	5’- GTTCTACCCGTGTATTGCTCTTTG-3’	-
ND_HN_Reg1	5’- CCCACAACATCCGTTCTACC-3’	5’- CGAAGCACACCAAGTGCTAA-3’	-
ND_HN_Reg2	5’- TTAGCACTTGGTGTGCTTCG-3’	5’- ACCGTGAGAATTCTGCCTTC-3’	-
ND_HN_Reg3	5’- TGAGGACCCAATGCTGACTA-3’	5’- CCCCGAATAGGGTATTGGAT-3’	-

qRT-PCR = Quantitative reverse transcription polymerase chain reaction

### RT-PCR and sequencing of F and HN genes

The samples that showed positive NDV and negative AIV results were subjected to RT-PCR to obtain PCR products for further sequencing. Four pairs of F gene primers and three pairs of overlapping HN gene primers were used (Table-2). Each pair of primers was used according to the manufacturer’s instructions (Invitrogen, Thermo Fisher Scientific). The RT-PCR reagent mix consisted of 2× the reaction mix, SuperScript™ III One-step RT-PCR with Platinum™ Taq (Invitrogen, Thermo Fisher Scientific), one pair of forward and reverse primers, NFW, and extracted RNA. Amplification was performed using Veriti™ (Thermo Fisher Scientific, USA) 96-well fast thermal cycler machine with the following conditions: RT (50°C for 30 min) for 1 cycle; pre-denaturation (94°C for 5 min) as much as 1 cycle; denaturation (94°C for 40 s), annealing (56°C for 30 s), elongation (72°C for 60 s) for 35 cycles, and post-extension (temperature 72°C for 4 min) for 1 cycle. The PCR products were then subjected to Sanger sequencing (Sanger Sequencing Services, 1^st^ Base, Malaysia).

### Sequence analysis

The sequences of each primer were assembled using MEGA-X software and then spliced into a single unit in each F and HN gene. The primers were designed based on the NDV_layer/Indonesia/BYL3-I11/982/2014 (acc no. MN557411) using the primer3 online tool. The available complete F and HN gene sequences of class II NDV isolates were downloaded from the GenBank of the National Center for Biotechnology Information. The sequences in each dataset were aligned with the NDV sequences of this study using the Multiple Alignment tool of the MEGA-X program.

The amino acid sequence of the F gene at the cleavage site was analyzed to determine the pathogenicity of the isolates. Genetic variations were observed in the nucleotide base sequences of the F and HN genes. The estimates of the average evolutionary distances between genetic groups were inferred using MEGA-X [19]. Groups including the most closely related viruses and representative viruses of class II and I as the out-group were aligned from the baseline dataset and analyzed further. Phylogenetic analyses were conducted using the maximum likelihood model and were constructed using 1000 bootstrap replicates [20]. The Roman numerals presented in the taxa names in the phylogenetic trees represent the respective genotypes for each isolate. Based on genetic variation, phylogenetic tree analysis and evolutionary distances between the different groups were used to classify the studied isolates.

### Virus isolation and identification

Eight collected samples and one live vaccine (LaSota) were placed in 1 mL PBS with penicillin G and streptomycin, each at a final concentration of 10,000 units, and then stored for 60 min at room temperature. The mixture was centrifuged at 1000 g for 5 min. Two hundred microliters of each supernatant and PBS (negative control) were inoculated separately into 10-day-old embryonated chicken eggs (ECE) that specific antibody negative. The inoculated ECE was incubated at 37°C for 5 days and observed for death every 24 h. Fresh allantoic fluid was harvested from infected eggs after the embryo died or after 120 h of inoculation if alive. The allantoic fluid was then subjected to hemagglutination (HA) and hemagglutination inhibition (HI) tests.

### Gross pathological, histopathological, and immunohistochemistry (IHC) examination

Embryos from inoculated chicken eggs were collected for gross pathological examination. Histopathological examination and IHC were conducted on selected tissues of the embryos inoculated with viruses of different pathotypes. They were then fixed with 10% NBF, processed, and embedded in paraffin. Sections measuring 4 mm in thickness were prepared and stained with hematoxylin and eosin. Duplicate sections were stained immunohistochemically. IHC staining included the use of primary rabbit anti-NDV polyclonal antibody (Bioss Antibodies, USA) in 1:500 dilution (Dako, S3022), secondary antibody Dako REAL™ envision™/HRP, rabbit/mouse (Dako North America, USA) (K5007), and Dako REAL™ DAB + chromogen in Dako REAL™ Substrate Buffer (Dako North America). After staining, samples were dripped with ultramounted Dako and placed on a hot plate at 70°C for 20 min. Furthermore, slides were mounted, cleared, and sealed with coverslips.

### Histopathological analysis of chicken embryos

Data on histopathological changes and distribution of NDV were analyzed descriptively. The severity of histopathological lesions such as necrosis, hemorrhage, and inflammatory cell infiltration was rated as low (focal), moderate (multifocal), and high (visible diffuse). The distribution of NDV was observed in various tissues via IHC staining. Analysis of immunopositivity in tissues was done using a scoring system of cells that showed positive reactions to immunohistochemical staining in each field of view. The scoring system was adapted from Etriwati *et al*. [21].

## Results

### NDV diagnosis using qRT-PCR

Molecular NDV diagnosis using qRT-PCR was done based on the amplification of the M gene for NDV screening. Eight samples were diagnosed with NDV without AIV. The identification results are presented in Table-1.

### RT-PCR and sequencing of the F and HN genes

The PCR products were sent for sequencing. The results of the ABI files were assembled, analyzed, and spliced using MEGA-X. The assembly results of the F and HN gene sequences (n = 16) from the eight NDVs obtained in this study were submitted to GenBank and are available with accession numbers as listed in Table-1.

### Sequence analysis

**Figure-1:**
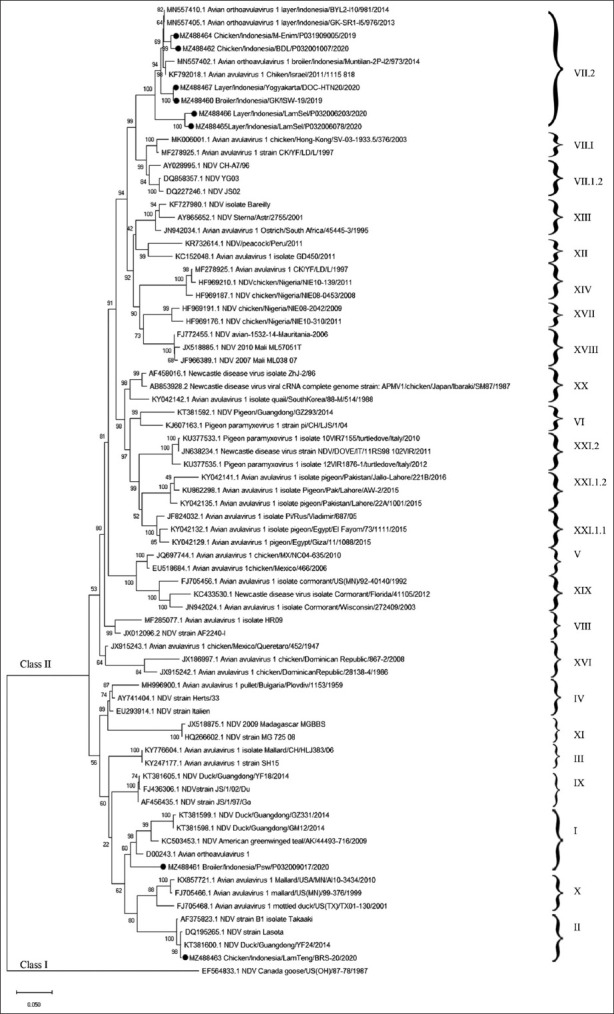
Phylogenic tree using the Maximum Likelihood method and Kimura 2-parameter model. This analysis involved 75 nucleotide sequences. There were a total of 1662 positions in the final dataset. Black dot (•) is the NDV isolate of this study. NDV=Newcastle disease virus.

Of the eight NDVs, six were determined to be virulent and two were non-virulent, based on the cleavage site analysis (Table-1). A phylogenetic analysis of the F gene revealed that the genotypes of the isolates varied. The velogenic strain was closely related to the previous Indonesian NDV isolate, namely, sub-genotype VII.2 (Figure-1). One of the lentogenic strains belonged to genotype I and the other to genotype II, which was close to the LaSota strain. The genetic distances of the nucleotide sequences between the samples in this study and another NDV are summarized in Table-3.

**Table 3:** Genetic distances based on the F gene between isolates in this study and NDV isolates from various genotypes from the data in GenBank.

S. No.	NDV	1	2	3	4	5	6	7	8	9	10	11	12	13	14	15
1.	KU377533.1_Pigeon_10VIR7155/turtledove/Italy/2010 (G_XXI.2)															
2.	KC433530.1_NDV_Cormorant/Florida/41105/2012 (G _XIX)	0.180														
3.	KY042142.1_quail/SouthKorea/88-M/514/1988 (G_XX)	0.101	0.147													
4.	JX915242.1_Avian_avulavirus_1_chicken/DominicanRepublic/28138-4/1986 (G_XVI)	0.157	0.180	0.134												
5.	JX518885.1_NDV_2010_Mali_ML57051T (G_XVIII)	0.153	0.167	0.116	0.162											
6.	HF969176.1_NDV_chicken/Nigeria/NIE10-310/2011 (G_XVII)	0.163	0.169	0.124	0.168	0.105									
7.	HF969210.1_NDVchicken/Nigeria/NIE10-139/2011 (G_XIV)	0.176	0.186	0.151	0.179	0.130	0.130									
8.	AY865652.1_NDV_Sterna/Astr/2755/2001 (G_XIII)	0.132	0.164	0.113	0.151	0.111	0.114	0.129								
9.	KC152048.1_Avian_avulavirus_1_isolate_GD450/2011 (G_XII)	0.143	0.167	0.112	0.150	0.103	0.113	0.125	0.093							
10.	JX518875.1_NDV_2009_Madagascar_MGBBS (G_XI)	0.206	0.217	0.176	0.199	0.204	0.196	0.237	0.195	0.208						
11.	KX857721.1_Avian_avulavirus_1_Mallard/USA/MN/AI10-3434/2010 (G_X)	0.187	0.198	0.165	0.165	0.180	0.179	0.203	0.178	0.167	0.184					
12.	KT381605.1_NDV_Duck/Guangdong/YF18/2014 (G_IX)	0.164	0.173	0.134	0.148	0.160	0.155	0.196	0.147	0.153	0.151	0.115				
13.	JX012096.2_NDV_strain_AF2240-I (G_VIII)	0.118	0.126	0.083	0.106	0.110	0.115	0.141	0.106	0.104	0.153	0.135	0.104			
14.	MN557410.1_Avian_orthoavulavirus_1_layer/Indonesia/BYL2-I10/981/2014 (G_VII.2)	0.135	0.162	0.105	0.151	0.115	0.120	0.139	0.097	0.104	0.196	0.165	0.150	0.098		
15.	KY042129.1_Avian_avulavirus_1_pigeon/Egypt/Giza/11/1088/2015 (G_XXI.1.1)	0.086	0.163	0.088	0.155	0.133	0.146	0.150	0.124	0.125	0.203	0.189	0.154	0.101	0.120	
16.	KT381592.1_NDV_Pigeon/Guangdong/GZ293/2014 (G_VI)	0.113	0.170	0.096	0.153	0.146	0.151	0.174	0.138	0.136	0.196	0.182	0.166	0.106	0.136	0.105
17.	JQ697744.1_Avian_avulavirus_1_chicken/MX/NC04-635/2010 (G_V)	0.149	0.095	0.115	0.135	0.136	0.133	0.148	0.128	0.130	0.178	0.161	0.131	0.088	0.134	0.133
18.	AY741404.1_NDV_strain_Herts/33 (G_IV)	0.135	0.153	0.108	0.119	0.134	0.138	0.164	0.126	0.129	0.117	0.110	0.074	0.078	0.121	0.131
19.	KY247177.1_Avian_avulavirus_1_strain_SH15 (G_III)	0.169	0.178	0.143	0.156	0.160	0.164	0.199	0.154	0.154	0.164	0.125	0.085	0.111	0.144	0.162
20.	DQ195265.1_NDV_strain_LaSota (G_II)	0.198	0.189	0.168	0.182	0.191	0.193	0.227	0.188	0.183	0.196	0.111	0.120	0.146	0.182	0.190
21.	D00243.1_Avian_orthoavulavirus_1 (G_I)	0.160	0.176	0.139	0.147	0.164	0.151	0.193	0.147	0.153	0.168	0.088	0.086	0.107	0.137	0.155
22.	EF564833.1_NDV_Canada_goose/US(OH)/87-78/1987 (Class I)	0.424	0.436	0.408	0.412	0.427	0.412	0.430	0.419	0.412	0.437	0.385	0.419	0.404	0.438	0.426
23.	MZ488467_Layer/Indonesia/Yogyakarta/DOC-HTN20/2020 (G_VII.2)	0.143	0.163	0.114	0.157	0.127	0.129	0.147	0.109	0.115	0.202	0.174	0.159	0.107	0.022	0.120
24.	MZ488466_Layer/Indonesia/LamSel/P032006203/2020 (G_VII.2)	0.160	0.179	0.126	0.164	0.149	0.143	0.175	0.138	0.142	0.203	0.158	0.135	0.117	0.068	0.139
25.	MZ488465Layer/Indonesia/LamSel/P032006078/2020 (G_VII.2)	0.160	0.176	0.127	0.171	0.152	0.149	0.176	0.138	0.142	0.206	0.156	0.133	0.122	0.065	0.136
26.	MZ488464_Chicken/Indonesia/M-Enim/P031909005/2019 (G_VII.2)	0.146	0.174	0.114	0.161	0.127	0.134	0.148	0.108	0.119	0.201	0.173	0.158	0.108	0.020	0.127
27.	MZ488463_Chicken/Indonesia/LamTeng/BRS-20/2020 (G_II)	0.196	0.191	0.166	0.183	0.190	0.193	0.225	0.187	0.185	0.196	0.111	0.121	0.144	0.181	0.189
28.	MZ488462_Chicken/Indonesia/BDL/P032001007/2020 (G_VII.2)	0.144	0.170	0.112	0.159	0.128	0.136	0.148	0.112	0.118	0.202	0.173	0.158	0.108	0.024	0.122
29.	MZ488461_Broiler/Indonesia/Psw/P032009017/2020 (G_I)	0.163	0.181	0.135	0.153	0.157	0.147	0.179	0.130	0.144	0.175	0.113	0.107	0.107	0.099	0.153
30.	MZ488460_Broiler/Indonesia/GK/ISW-19/2019 (G_VII.2)	0.144	0.163	0.115	0.159	0.128	0.131	0.149	0.110	0.117	0.202	0.175	0.159	0.108	0.022	0.122

**S. No.**	**NDV**	**16**	**17**	**18**	**19**	**20**	**21**	**22**	**23**	**24**	**25**	**26**	**27**	**28**	**29**	**30**

1.	KU377533.1_Pigeon_10VIR7155/turtledove/Italy/2010 (G_XXI.2)														
2.	KC433530.1_NDV_Cormorant/Florida/41105/2012 (G _XIX)															
3.	KY042142.1_quail/SouthKorea/88-M/514/1988 (G_XX)															
4.	JX915242.1_Avian_avulavirus_1_chicken/DominicanRepublic/28138-4/1986 (G_XVI)															
5.	JX518885.1_NDV_2010_Mali_ML57051T (G_XVIII)															
6.	HF969176.1_NDV_chicken/Nigeria/NIE10-310/2011 (G_XVII)															
7.	HF969210.1_NDVchicken/Nigeria/NIE10-139/2011 (G_XIV)															
8.	AY865652.1_NDV_Sterna/Astr/2755/2001 (G_XIII)															
9.	KC152048.1_Avian_avulavirus_1_isolate_GD450/2011 (G_XII)															
10.	JX518875.1_NDV_2009_Madagascar_MGBBS (G_XI)															
11.	KX857721.1_Avian_avulavirus_1_Mallard/USA/MN/AI10-3434/2010 (G_X)															
12.	KT381605.1_NDV_Duck/Guangdong/YF18/2014 (G_IX)															
13.	JX012096.2_NDV_strain_AF2240-I (G_VIII)															
14.	MN557410.1_Avian_orthoavulavirus_1_layer/Indonesia/BYL2-I10/981/2014 (G_VII.2)															
15.	KY042129.1_Avian_avulavirus_1_pigeon/Egypt/Giza/11/1088/2015 (G_XXI.1.1)															
16.	KT381592.1_NDV_Pigeon/Guangdong/GZ293/2014 (G_VI)															
17.	JQ697744.1_Avian_avulavirus_1_chicken/MX/NC04-635/2010 (G_V)	0.141														
18.	AY741404.1_NDV_strain_Herts/33 (G_IV)	0.136	0.112													
19.	KY247177.1_Avian_avulavirus_1_strain_SH15 (G_III)	0.169	0.138	0.072												
20.	DQ195265.1_NDV_strain_LaSota (G_II)	0.188	0.166	0.117	0.126											
21.	D00243.1_Avian_orthoavulavirus_1 (G_I)	0.167	0.143	0.079	0.090	0.103									
22.	EF564833.1_NDV_Canada_goose/US(OH)/87-78/1987 (Class I)	0.424	0.412	0.406	0.396	0.412	0.399									
23.	MZ488467_Layer/Indonesia/Yogyakarta/DOC-HTN20/2020 (G_VII.2)	0.143	0.140	0.133	0.155	0.189	0.147	0.432								
24.	MZ488466_Layer/Indonesia/LamSel/P032006203/2020 (G_VII.2)	0.159	0.154	0.124	0.141	0.129	0.125	0.432	0.082							
25.	MZ488465Layer/Indonesia/LamSel/P032006078/2020 (G_VII.2)	0.157	0.154	0.128	0.141	0.129	0.124	0.422	0.054	0.028						
26.	MZ488464_Chicken/Indonesia/M-Enim/P031909005/2019 (G_VII.2)	0.150	0.145	0.129	0.155	0.195	0.146	0.436	0.038	0.083	0.079					
27.	MZ488463_Chicken/Indonesia/LamTeng/BRS-20/2020 (G_II)	0.190	0.165	0.117	0.126	0.004	0.101	0.412	0.188	0.128	0.127	0.193				
28.	MZ488462_Chicken/Indonesia/BDL/P032001007/2020 (G_VII.2)	0.147	0.141	0.129	0.152	0.194	0.147	0.444	0.041	0.087	0.083	0.022	0.192			
29.	MZ488461_Broiler/Indonesia/Psw/P032009017/2020 (G_I)	0.163	0.145	0.094	0.112	0.139	0.068	0.402	0.114	0.140	0.142	0.115	0.136	0.115		
30.	MZ488460_Broiler/Indonesia/GK/ISW-19/2019 (G_VII.2)	0.145	0.140	0.135	0.157	0.191	0.148	0.435	0.001	0.084	0.055	0.038	0.190	0.041	0.116	

NDV=Newcastle disease virus

### Virus isolation and identification

Ten embryonic chicken eggs were inoculated with eight organs or cloacal swab samples, one with vaccine strain, and one with PBS as a negative control. All samples were analyzed using HA and HI tests. The results showed that the titer of viral HA ranged from 2^1^ to 2^7^, which could be inhibited by standard NDV-positive serum. Negative HA-HI results were obtained from two samples with Ct values >30, as well as from the negative control. Samples with varying Ct values showed varying viral titers. The lower the Ct value, the higher the isolated virus titer. Samples with a Ct value above 30 showed negative results in the first isolation and required passage in the ECE more than two times for positive results. Several isolates were passaged repeatedly to obtain a uniform titer of 2^6^, which was used in the treatment of macroscopic and microscopic observations of embryonic lesions after NDV inoculation. Based on the time of embryonic death after inoculation, there were six isolates, including velogenic strains, and two samples, including lentogenic strains.

### Gross pathological, histopathological, and IHC examination

**Figure-2:**
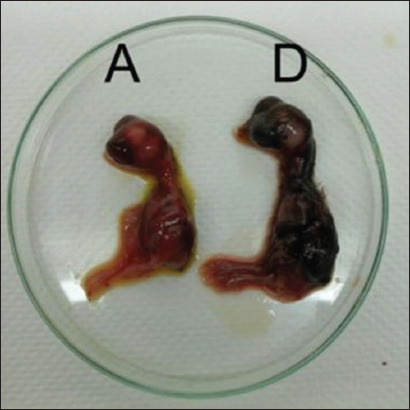
Chicken embryos 2 days post-inoculation, A=ECE infected by velogenic strain NDV and D=negative control. NDV=Newcastle disease virus.

A gross examination was conducted after the death of the infected embryo. The dead embryos were hemorrhagic, had no feathers, and were stunted from virulent NDV infection (Figure-2). Hemorrhages were observed in almost all visceral organs, but not in detail because the organs were too small. No gross lesions were observed in the two non-virulent NDV and negative control embryos. Histopathological lesions in the tissues are summarized in Table-4. Viral antigens have been demonstrated in many embryonic organs.

**Table 4:** Results of histopathologic analysis of lesions and distribution NDV in tissues of ECE.

Tissue	Necrosis				Hemorrhage				Inflammatory cell infiltration				Distribution/expression			
			
	A	B	C	D	A	B	C	D	A	B	C	D	A	B	C	D
Brain	+++	-	-	-	+++	+	+	-	++	-	-	-	++/++	++/++	-	-
Trachea	++	+	+	-	++	+	++	-	++	++	+++	-	+++/+++	+++/+++	+++/+++	-
Lungs	+++	+	+	-	+++	+	+	-	+++	+	+	-	+++/+++	+++/+	+++/+++	-
Proventriculus	+++	+	+	-	++	+	++	-	++	+	++	-	+++/+++	+++/++	++/+++	-
Intestinum	+++	+	-	-	++	+	+	-	++	+	-	-	+++/+++	+++/+++	-	-
Heart	+	-	-	-	+++	++	++	-	++	-	-	-	+++/+++	++/++	-	-
Kidney	+++	-	++	-	+++	+	++	-	+++	+	+	-	+++/+++	+++/++	++/++	-
Liver	++	++	-	-	+++	++	++	-	++	-	-	-	+++/+++	+++/+++	-	-
Skin	+++	-	-	-	+++	+	+	-	+++	-	-	-	+++/+++	+++/+++	-	-

NDV = Newcastle disease virus, A = NDV Velogenic, B = NDV Lentogenic, C = NDV vaccine strain LaSota and D = Negative control. Lesion (+) = focal, (++) = multifocal, and (+++) = diffuse. IHC staining (+) = low, (++) = moderate, and (+++) = severe

## Discussion

The qRT-PCR of the NDV and AIV Matrix genes was performed to diagnose the specimens from commercial and native chickens. With or without symptoms, both chickens contained NDV particles without AIV. NDV was identified using the qRT-PCR method for the M gene [22]. This method can only be used to identify the presence of RNA from APMV-1 [23]; thus, it cannot be used to confirm the NDV strain. According to Office International des Epizooties [4], a specific primer for the F gene covering the cleavage site is used for the molecular identification of NDV. The qRT-PCR method had more sensitivity for NDV detection than the RT-PCR. The F gene primer–probe set used in qRT-PCR was able to detect 10^2^-10^4^ copies of RNA and at least 10 NDV particles [22]. The minimum concentration of ND viral RNA required for RT-PCR with primer F was 10^5^ [24].

The Ct value of the sample in this study was between 18.09 and 32.52. Samples with a Ct value below 25 can result in a viral titer of 2^3^-2^7^. However, samples with Ct values above 30 require passage of up to more than two times to obtain a positive result. Similarly, Munster *et al*. [25] reported a correlation between the number of viral RNA copies and the ability to isolate viruses from swab samples in the field.

Eight NDVs in this study, based on phylogenic tree analysis, belonged to three different genotypes. NDV genotype VII is circulating in chickens and other birds in Indonesia and other countries. Genotype VII has been reported to cause outbreaks in commercial and backyard chickens in Indonesia [26]. The NDV genotype I was isolated from a live bird market [27]. ND caused by NDV genotypes II, VI, and VII was reported in ducks in East Java, Indonesia [28]. The sub-genotype VII.1.2, reported in fowls, pigeons, and geese in China, as well as in chickens in Pakistan, Israel, Iran, and Zimbabwe, from the analysis caused the alteration and update of the phylogenetic classification of NDV [2]. Numerous variations of the NDV isolated from different wild animals constitute transmission information between wild and domestic birds that can transmit the virus to the poultry industry, or vice versa [29]. The crucial part of this study was the identification of the NDV genotype I in commercial chicken, genotype II in native chicken, and genotype VII sub-genotype VII.2 in both.

Amino acid sequences at the cleavage site of the isolates were compared to NDV data from GenBank. There were differences in amino acid composition. Five NDVs in this study, with a black dot mark, have ^112^RRQKR↓F^117^ on the cleavage site. This pattern is similar to the Indonesian NDV isolated from cockatoo in 1990 (AY562985), Boyolali (MN557410.1); broiler chickens from Muntilan in 2014 (MN557401.1); and NDV in commercial chickens in Bogor in 2011 [16]. The cleavage site pattern of one NDV from the layer was ^112^RRRKR↓F^117^. There is a substitution from 114Q to 114R. This pattern is similar to that of NDV in chickens from Nigeria (HF969176). The amino acid sequence at the cleavage site that is pathogenic to chickens is ^112^R/K-R-Q/K/R-K/R-R^116^ and phenylalanine ^117^F [30, 31]. Lien *et al*. [32] reported that there was no significant difference between three, four, and even five basic amino acids in the cleavage site with higher pathogenicity. Isolates with a cleavage site pattern of ^112^RRQKRF^117^, ^112^RRQRRF^117^, and ^112^RRRKRF^117^ did not show a significant difference in the ICPI and IVPI tests.

The virulence and pathogenicity of NDV can be determined based on the analysis of the amino acid sequence at the cleavage site and MDT. Six NDVs in this study were virulent and velogenic, based on the cleavage site and MDT <60 h. There were two NDVs in this research, which had leucine at position 117. The LaSota strain, which has an amino acid sequence at the cleavage site ^112^GRQGR↓L^117^, is a lentogenic NDV [4]. One NDV from the backyard had the same pattern as LaSota strain, which is a vaccine strain used in poultry farms in Indonesia. However, one NDV from the broiler flock has a substitution from R^113^ to K^113^. The pattern ^112^GKQGR↓L^117^ is similar to NDV genotype I from ducks in China (KT381598).

All NDVs in this study had conserved cysteine residues. The fusion of NDV revealed 12 conserved cysteine residues at positions 25, 76, 199, 338, 347, 362, 370, 394, 399, 401, 424, and 523 [33]. A comparison of the predicted amino acid sequences of the NDV at the fusion protein showed that there were seven neutralization epitopes located at amino acids 72, 74, 75, 78, 79, 151-171, and 343 [33, 34]. Almost all isolates from this study had a conserved residue, except Broiler_A032009017, which had a substitution from D^170^ to N^170^.

The stalk of the HN protein directly interacted with the F protein. The HN–F interaction was mediated by a stretch of the conserved amino acid heptad repeats (HRA from 74 to 88 and HRB from 96 to 110) in the HN protein [7]. The nucleotide sequence HRA of the lentogenic NDV was conserved at this site and is consistent with the consensus on NDV LaSota strain, in contrast to velogenic NDV strains, which had three substitutions at positions 74, 75, and 77 in HRA. The lentogenic NDV strain in Broiler_A032009017 had one substitution at ^101^T to ^101^S, while two substitutions were seen in all velogenic NDV strains: ^101^T to ^101^S and ^102^T to ^102^I. The HN protein has four amino acids (174 R, 401E, 416R, and 526Y) that are essential for the binding of the receptor to the host. The amino acid at this position remained conserved. The hemagglutinating activities of HN proteins are influenced by three regions, namely: ^234^NRKSCSV^240^, ^314^FPVYGGL^320^, and ^399^GAEGRIL^405^. There was no modification in the velogenic NDV strain, but there was a substitution at ^315^P to ^315^S in BRS_20 (lentogenic like LaSota). The neutralization epitopes of NDV were not modified at positions ^193^LSGCRDHSH^201^, ^263^K, ^287^D, ^321^K, ^332^GK^333^, ^345^LDEQDYQIR^353^, ^356^K, ^481^N, ^494^D, and ^513^RVTRVSSSS^521^. The NDVs in this study had variations in the antigenic site (^98^N, ^494^D, ^514^V) between velogenic and lentogenic strains. This is similar to the previous Indonesian NDVs and NDV genotype VII seen in Iran [^12^, ^35^]. All lentogenic isolates showed two substitutions: ^263^K to ^263^N and ^494^G to ^494^D, similar to the consensus sequence of the LaSota vaccine [36, 37]. The presence of antigenic variants and escape antibody neutralization may be due to mutations in the neutralizing epitopes of the HN proteins of NDVs [13, 33].

There are 13 cysteine residues that are highly conserved at positions 123, 172, 186, 196, 238, 247, 251, 344, 455, 461, 465, 531, and 542 on the HN protein [36, 38, 39]. The HN gene analysis of NDV from the velogenic strain revealed conserved cysteine residues. The cysteine residue at position 123 is one of the amino acid positions that are variable in the LaSota strain and the NDV genotype II (tryptophan/W), as shown in sample BRS_20. Cysteine plays a role in stabilizing the HN protein structure. The cysteine residue of amino acid position 123 was specifically found in virulent NDV [10], which is similar to the virulent isolates in this study. The HN protein of this isolate contained six potential N-glycosylation sites (N-X-S/T) at positions 119, 341, 433, 481, and 538, which were conserved, except for position 508 (D-I-S), for velogenic samples from native chicken, and at position 119 (N-S-S) from Broiler_A03200901. The role of N-linked carbohydrates in the activity of the HN glycoprotein includes cell attachment. The glycosylation site of the HN gene plays an important role in increasing the affinity for receptors on target cells and is involved in influencing the virulence of NDV [40]. An analysis of the amino acid polymorphism site of the HN protein showed that the NDV isolates in this study varied depending on the type of strain. The NDV isolated from Broiler_A032009017 had many variations compared to other isolates and was slightly different from samples from lentogenic strains because they belonged to different genotypes. NDV with the E347K variation in HN protein in the cross-neutralization assay showed a lower antigenic association with LaSota [41]. In this study, none of the isolates had mutations in the E347K amino acid residue, as previously reported in Indonesia [42].

The value of genetic distance in the F gene between velogenic isolates in this study was between 0.1% and 8.7% (Table-3) and the homology value was between 99.9% and 91.3%. The genetic distance with the previous Indonesian isolates was between 2.2% and 6.8%, with a homology value of 97.8-93.2%. The NDV in this study had varied genetic distances between lentogenic and velogenic strains. The differences between the velogenic strain and BRS_20 (genotype II) and Broiler_A03200901 (genotype I) ranged from 12.9% to 19.4% and from 11.4% to 14.2%, respectively. The genetic distance among the NDV lentogenic strains in this study was 13.6%. The genetic distance value of the isolate from BRS_20 compared to the LaSota strain has a genetic distance value of 0.4% and a homology value of 99.6% (Table-3). All velogenic NDVs in this study still had a genetic distance that is close to the previously reported NDV from chickens in Indonesia. The NDV LaSota strain is different from field viruses circulating in Indonesia [43] and China [44]. However, there is one lentogenic NDV from native chickens close to LaSota used as a vaccine strain in Indonesia. The lentogenic NDV strain circulating in native chickens might contribute to outbreaks in East Java, Indonesia. NDV-infected native chickens can act as reservoirs and contribute to outbreaks in poultry [17].

The pathogenesis of NDV in chicken embryos can be observed in the distribution of lesions and NDV in chicken embryo tissues. The dose used for infection in ECE was 0.2 mL with a viral content of 2^6^ HAU per ECE. The infective dose of ECEs is a viral titer of 2^3^, whereas that of the chicken is 2^6^ [45]. There was no significant difference in the timing of embryo death and gross lesions of NDV infection with a viral dose of 2^6^-2^10^ [46]. Embryos inoculated with NDVs that have a virulent pathotype die in <60 h and show clinical signs such as bleeding, hairlessness, and stunting. These results are consistent with the molecular characterization of the six virulent NDVs in this study. Meanwhile, embryos inoculated with non-virulent NDV did not die until the 5^th^ day of observation. This is consistent with the molecular characterization of the two non-virulent NDVs from the cleavage site analysis.

**Figure-3:**
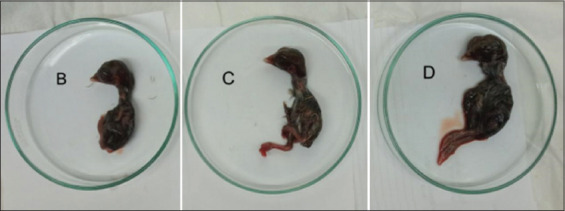
Chicken embryos 5 days post-inoculation, B=ECE infected by Lentogenic strain NDV, C=ECE infected by vaccine LaSota and D=Negative control. NDV=Newcastle disease virus.

Observations of histological changes and distribution of NDV were carried out on samples of the NDV strains, velogenic, lentogenic, and LaSota, as a comparison of vaccines and negative controls. Histological lesions have been observed in various embryonic tissues of the respiratory, digestive, circulatory, urinary tract, and nervous systems, and embryonic skin [47, 48], as well as in organs of adult chickens [5]. Velogenic NDV infection shows clinical signs of hemorrhage and necrosis of the respiratory tract and digestive tract and causes lesions in the nervous system [47]. NDV infection in ECE is systemic and NDV appears to proliferate in various embryonic cells, as observed via IHC staining within 48 h post-infection [49]. Lentogenic NDV-inoculated embryos were collected after 5 days, and there were no macroscopic lesions observed. The embryos were observed to be normal in the negative control (Figure-3). Histological observation of the tissue showed mild hemorrhage in almost all organs, and necrosis and inflammatory cell infiltration were only observed lightly in the respiratory, digestive, and renal organs.

The lungs and trachea as respiratory organs were observed in every chicken embryo. In this study, tissue damage in chicken embryos appears to be severe because of age and immune system maturity factors. Hemorrhage and necrosis were more evident in the lungs and trachea of embryos with velogenic NDV infection than in those with lentogenic infection. Inflammatory cell infiltration was observed under all conditions. NDV distribution was observed in the lungs and trachea after treatment with the two NDV strains. There was no visible change or positive immunoreaction in the negative control group. Immunopositive reactions in the lungs and trachea of chicken embryos have also been reported by Astawa and Adi [50]. The distribution of the NDV strain of LaSota after infection in chickens can also be seen in the nasal mucosa [51].

**Figure-4:**
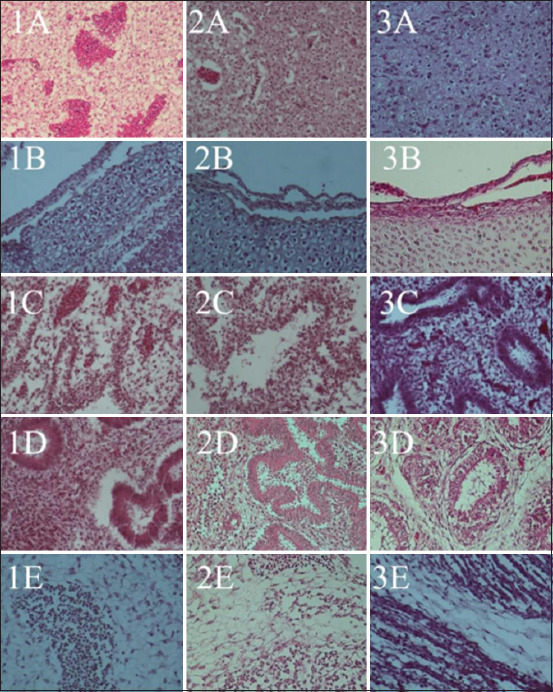
Hematoxylin and eosin staining of chicken embryo tissue inoculated with NDV. 1=Velogenic strain, 2=Lentogenic strain, 3=no infection as a negative control. Organs A=Brain, B=Trachea, C=Lungs, D=Proventriculus, E=Skin. NDV=Newcastle disease virus.

NDV infection and replication in chickens cause neurological disorders due to nerve cell damage in the brain. The characteristic lesion of the brain due to NDV infection is perivascular cuffing (Figure-4). Hemorrhage, necrosis, and inflammatory cell infiltration are also observed in the brain after velogenic NDV infection. The characteristic lesions in the embryonic brain after NDV infection are congestion, hemorrhage, and inflammation [50]. Inflammation begins with spreading macrophages in the perivascular cuff and with the eventual spreading to astrocytes and microglia in the brains of chickens infected with NDV; inflammatory cells and astroglia were previously found [21, 52] in the chicken embryo brain [50]. Mild hemorrhage, no necrosis, and inflammatory cell infiltration are seen in a lentogenic NDV infection. The distribution of NDV in the brain occurred in velogenic and lentogenic NDV infections in this study but not in the vaccine inoculation. Moura *et al*. [53] reported a minimal spread of replication in the ependymal layer of the brain during lentogenic infection.

**Figure-5:**
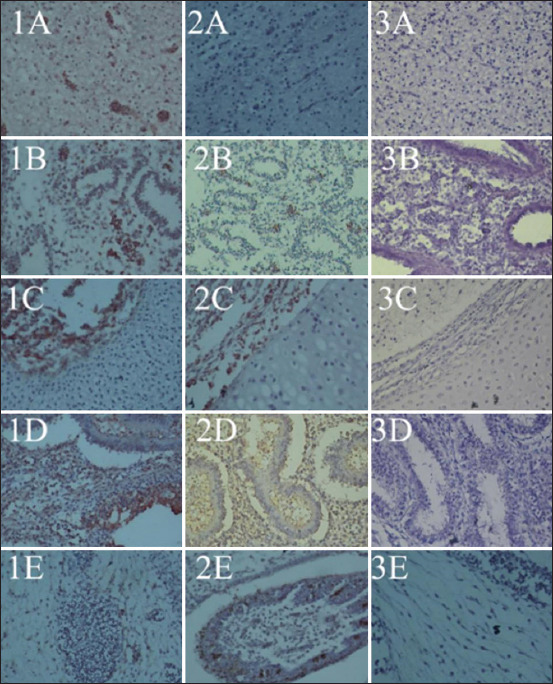
Immunohistochemistry staining of chicken embryo tissue inoculated with NDV. 1=Velogenic strain, 2=Lentogenic strain, 3=No infection as a negative control. Organs A=Brain, B=Lungs, C=Trachea, D=Proventriculus, E=Skin. NDV=Newcastle disease virus.

Histopathological examination revealed hemorrhage and inflammatory cell infiltration of the skin. There was an immunopositive reaction to NDV, as seen through IHC staining of the sebaceous tissue of the skin and feather follicle (Figure-5). This is similar to the study of Al-Garib [49], who stated that NDV in chicken embryo skin was stained with IHC at 24 h after virulent NDV infection and 72 h after avirulent NDV infection.

The histopathological features of the intestine of chicken embryos aged 11 days showed necrosis up to the erosion of the mucosal epithelial and muscular layers. Replication of NDV in intestinal lymphoid follicles causes hemorrhage and edema in the internal organs due to vascular disorders. There was an immunopositive reaction to NDV in necrotic areas, as seen using IHC staining. The distribution of NDV in digestive organs is according to that reported in the tissues of adult chickens infected with velogenic NDV [21, 54, 55]. The distribution of NDV in various organs is also seen in lentogenic strains (Figure-5) but does not cause tissue damage as in velogenic NDV inoculation. Bwala *et al*. [56] stated that the distribution of the post-vaccination lentogenic strain NDV can also be stained with IHC for reproductive organs.

NDV was detected in lesions of various organs, and they were found primarily intracytoplasmic and rarely in the nucleus of the affected cells. The lentogenic NDV was observed in almost all organs, although there was no severe organ damage, as in the case of velogenic NDV. This proves that the lentogenic NDV also reaches the digestive and respiratory organs but does not cause significant damage; thus, embryo death does not occur.

## Conclusion

Based on the F gene characterization, the velogenic strain NDV (genotype VII.2) and lentogenic strain NDV (genotypes I and II) were found in commercial and backyard chickens. The neutralization epitope and antigenic site on the HN gene in all isolates were conserved, as in the previous circulating ND isolates in Indonesia, except for the lentogenic strain isolates, which varied at positions 263 and 494. The cysteine residues in the F and HN genes were conserved. The lentogenic strain NDV was distributed in almost all organs, especially the respiratory and digestive organs, but did not cause severe damage as did the velogenic strain NDV. The NDV in this study is relevant to the conditions reported in the field. Velogenic strains circulating in commercial and domestic chickens showing clinical symptoms varied with death. Lentogenic strains were found in chickens that did not show any clinical symptoms. The important information in this study is also related to reports of NDV genotype I in vaccinated grandparent stock broilers.

## Authors’ Contributions

LA: Performed the laboratory works, collected and analyzed the data, molecular analysis, computational analysis, and drafted and revised the manuscript. YPK: Supervised the study, verified the histological examination, and reviewed the manuscript. WA: Supervised the research, verified the virology detection, and reviewed the manuscript. MHW: Conceived the idea and designed the study, supervised the study and analysis, verified the molecular detection, and reviewed the manuscript. All authors have read and approved the final manuscript.
